# Multiple sclerosis lesion segmentation: revisiting weighting mechanisms for federated learning

**DOI:** 10.3389/fnins.2023.1167612

**Published:** 2023-05-18

**Authors:** Dongnan Liu, Mariano Cabezas, Dongang Wang, Zihao Tang, Lei Bai, Geng Zhan, Yuling Luo, Kain Kyle, Linda Ly, James Yu, Chun-Chien Shieh, Aria Nguyen, Ettikan Kandasamy Karuppiah, Ryan Sullivan, Fernando Calamante, Michael Barnett, Wanli Ouyang, Weidong Cai, Chenyu Wang

**Affiliations:** ^1^School of Computer Science, The University of Sydney, Sydney, NSW, Australia; ^2^Brain and Mind Centre, The University of Sydney, Sydney, NSW, Australia; ^3^Sydney Neuroimaging Analysis Centre, Camperdown, NSW, Australia; ^4^School of Electrical and Information Engineering, The University of Sydney, Sydney, NSW, Australia; ^5^NVIDIA Corporation, Singapore, Singapore; ^6^School of Biomedical Engineering, The University of Sydney, Sydney, NSW, Australia; ^7^Sydney Imaging, The University of Sydney, Sydney, NSW, Australia

**Keywords:** deep learning, federated learning, multiple sclerosis, segmentation, MRI

## Abstract

**Background and introduction:**

Federated learning (FL) has been widely employed for medical image analysis to facilitate multi-client collaborative learning without sharing raw data. Despite great success, FL's applications remain suboptimal in neuroimage analysis tasks such as lesion segmentation in multiple sclerosis (MS), due to variance in lesion characteristics imparted by different scanners and acquisition parameters.

**Methods:**

In this work, we propose the first FL MS lesion segmentation framework via two effective re-weighting mechanisms. Specifically, a learnable weight is assigned to each local node during the aggregation process, based on its segmentation performance. In addition, the segmentation loss function in each client is also re-weighted according to the lesion volume for the data during training.

**Results:**

The proposed method has been validated on two FL MS segmentation scenarios using public and clinical datasets. Specifically, the case-wise and voxel-wise Dice score of the proposed method under the first public dataset is 65.20 and 74.30, respectively. On the second in-house dataset, the case-wise and voxel-wise Dice score is 53.66, and 62.31, respectively.

**Discussions and conclusions:**

The Comparison experiments on two FL MS segmentation scenarios using public and clinical datasets have demonstrated the effectiveness of the proposed method by significantly outperforming other FL methods. Furthermore, the segmentation performance of FL incorporating our proposed aggregation mechanism can achieve comparable performance to that from centralized training with all the raw data.

## 1. Introduction

Multiple sclerosis (MS) is a chronic inflammatory and degenerative disease of the central nervous system, characterized by the appearance of focal lesions in the white and gray matter that topographically correlate with an individual patient's neurological symptoms and disability. Globally there are an estimated 2.3 million people with MS and, besides trauma, the disease constitutes the most common cause of neurological disability in young adults (Prinster et al., 2006; Coles et al., 2008; Plantone et al., 2015; Mills et al., 2018). Lesion characteristics, such as number and volume, are principal imaging metrics for both MS clinical trials and monitoring of the disease in clinical practice (Carass et al., 2017; Filippi et al., 2019; Schwenkenbecher et al., 2019; Pontillo et al., 2021). To this end, automatic, robust, and accurate MS lesion segmentation with Magnetic Resonance (MR) imaging is crucial to both MS research and patient management (Zijdenbos et al., 2002; Lladó et al., 2012; Brosch et al., 2016; Aslani et al., 2019; Cerri et al., 2021).

In classical MS lesion segmentation methods, the brain tissues types, such as white matter (WM), gray matter (GM), and cerebrospinal fluid (CSF), are firstly segmented based on the raw MR images via statistical methods, e.g., the Expectation-Maximization (EM) algorithm (Catanese et al., 2015; Beaumont et al., 2016) or Gaussian Mixture Modeling (Doyle et al., 2016; Knight and Khademi, 2016). Then, lesions are detected as outliers based on the tissue masks (Catanese et al., 2015; Beaumont et al., 2016; Doyle et al., 2016; Knight and Khademi, 2016). With the advent of deep learning-based medical data computing (Plis et al., 2014; Livne et al., 2019; Sun et al., 2019), deep learning models that learn representative features via convolutional modules have been widely employed for automatic MS lesion segmentation, achieving competitive performance (Brosch et al., 2016; Ghafoorian et al., 2017; Valverde et al., 2017; Wang et al., 2018; Zhang et al., 2018; Aslani et al., 2019; McKinley et al., 2020; Nair et al., 2020; Isensee et al., 2021; Ma et al., 2022).

Despite this, there remain significant challenges in the current methods (Danelakis et al., 2018; Ma et al., 2022). In clinical practice, the data quality of brain MRI varies across MRI scanners due to variance in image geometry, resolution, tissue intensity, and contrast conferred by differences in hardware (scanner and coil) and acquisition protocols (Kamnitsas et al., 2017; Dewey et al., 2019; Valverde et al., 2019; Ackaouy et al., 2020). These domain differences limit the performance of supervised learning methods when applied to images from new scanners (Kamnitsas et al., 2017; Ackaouy et al., 2020; Ma et al., 2022). Such phenomenon is referred to as the domain shift issue, which exists in various medical image analyses applications for multiple datasets from different resources (e.g, modalities, sites) (Valverde et al., 2019; Chen et al., 2020; Liu et al., 2020). Recently, cross-domain MS lesion segmentation methods have been further explored to enhance the models' generalization ability. In particular, the domain differences are alleviated by inducing the model to generate scanner-invariant features (Kamnitsas et al., 2017; Ackaouy et al., 2020), learning from synthetic images that follow the distribution of the target scanners (Palladino et al., 2020), and cross-scanner data harmonization (Dewey et al., 2019). A crucial prerequisite of these methods is that all the data from multiple scanners should be fed into the framework simultaneously. However, sharing clinical data across sites invokes privacy issues, which limit the practical applications of these methods in large collaborative studies (Li et al., 2020b; Guo et al., 2021).

Federated learning (FL) techniques where training is decentralized were proposed for multi-center computer vision while preserving data privacy and security (McMahan et al., 2017; Li et al., 2020a, 2021). Briefly, at the beginning of the FL process, each participating client is firstly assigned an initialized model. Note that throughout the paper, we use the notion “client” to represent the data in each distinct scanner or clinical center. Next, these models are trained using the local data in each client. After several training iterations, each client is required to share their private model weights with a central server, which aggregates these local weights and distributes them back to each client. Initialized by the updated weights from the server, the model in each client continues their local training for another round of FL process. By enriching the knowledge learned in each local model *without* sharing the raw data, the server side can eventually obtain a model for each client which can achieve a good performance simultaneously. FL methods have also been widely employed for multi-client medical image analysis (Li et al., 2020b; Guo et al., 2021; Liu et al., 2021a; Shen et al., 2021). In Li et al. (2020b) and Guo et al. (2021), each local model is incorporated with an adversarial domain discriminator to alleviate the inter-client distribution bias. However, the intermediate features in each local client are required to be shared across clients. Despite these privacy-preserving strategies, distributing features still incur the risk of data leakage. To solve this problem, FedBN (Li et al., 2021) has been proposed for domain adaptive FL by only processing the parameters outside the batch normalization layers of each local model.

**Figure 1:**
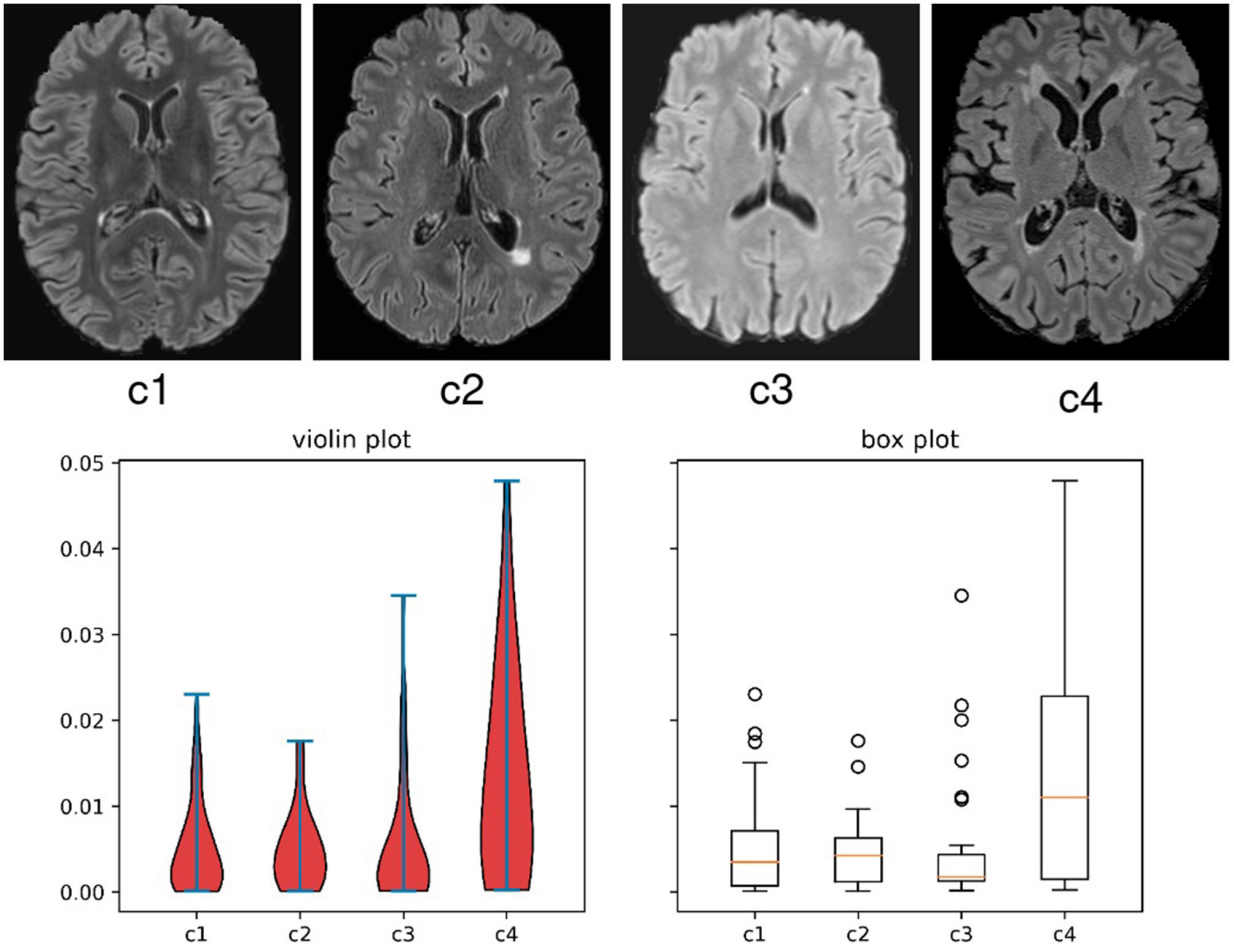
Evidence of the variance on appearance and lesion volume in multi-client studies in scenario 2 of this work, where cases are from clinical trials. The **top** images are examples of 2D slices from each client in the study. The **bottom** graphs are the violin and box plots for the lesion volume to brain volume ratio distributions per client for all the subjects in this FL study.

Although FL methods are effective to address these concerns in many medical imaging scenarios, their applicability is limited to MS lesion segmentation. Particularly, they have not considered the weighting strategies for the global aggregation and local training, which is crucial for FL MS segmentation. First, during aggregation, the central server averages the model parameters from all the local clients, assuming each local model has the same importance and performance. For MS lesion segmentation, the datasets from multiple clients, their data distribution and the lesion morphology and signal characteristics can vary greatly (Kamnitsas et al., 2017; Ackaouy et al., 2020), which can lead to divergence of the private local models, thereby conferring distinct segmentation characteristics when they are aggregated in the central server. By fusing a model with inferior segmentation performance to others with superior ability, the segmentation performance for the entire updated model may be compromised (Shen et al., 2021). Second, differences in the clinical distribution of patients can impact lesion burden, size, and morphology at a client level, generating significant inter-site variance in multi-client studies, as shown in Figure 1. As explored in Nichyporuk et al. (2021); Shirokikh et al. (2020), a model trained on a dataset with smaller lesions will usually present a lower performance due to the lack of lesion samples for training. However, the task loss functions in each client are optimized with the same importance in previous FL methods (McMahan et al., 2017; Li et al., 2020b, 2021), which would induce the inferior performance of the central model on the clients with smaller lesion sizes, and further influence the overall FL segmentation accuracy.

To solve the aforementioned issues, we propose a Federated MS lesion segmentation framework based on two dynamic Re-Weighting mechanisms (FedMSRW). Our FedMSRW method can alleviate the cross-client data distinctions caused by both image distributions and label variance. Specifically, we first alleviate the negative influence from the domain shift on the MRI data from different clients, by employing aggregation mechanisms from FedBN (Li et al., 2021). Second, during the model aggregation process, the model parameters from each client are assigned a weight based on their segmentation abilities during local training, including the segmentation performance and confidence. Models with higher abilities are assigned a higher weight and vice versa. To solve the lesion volume imbalance across different clients, we further propose to re-weight the task loss function in each client based on the average case-wise lesion volume ratio, i.e., the ratio of lesion volume to the brain volume, of the training data for that client. Motivated by Shirokikh et al. (2020), where more attention should be paid to smaller lesion objects during model training, the weights for the overall loss functions in clients with a smaller lesion volume are enlarged, and vice versa.

The major contributions of this work are summarized as follows:

- To the best of our knowledge, this work is the first application of privacy-preserving FL methods to the task of MS lesion segmentation and, in particular, to multi-client MS datasets featured with different data characteristics.
- We propose uncertainty-aware re-weighting mechanisms during the central model aggregation process to prevent the negative influence of the inferior local models.
- We further propose to re-weight the segmentation loss functions in each local client/center based on its local lesion volume ratio, addressing the impact of client-specific lesion variance in the multi-client MS datasets.
- We have conducted extensive experiments in two FL MS lesion segmentation scenarios using both public and real-world clinical MS datasets. Our FedMSRW method outperforms typical FL methods significantly.

## 2. Materials and methods

### 2.1. Datasets description

In this work, we have conducted experiments on two FL MS lesion segmentation scenarios. We first conduct experiments on a public MS lesion segmentation dataset from multiple clients, in favor of reproducibility. Second, we conduct experiments using our own multi-site MS lesion segmentation from different hospitals labeled following clinical trial standard, to further demonstrate the effectiveness of our proposed method in clinical practice. The study is approved by the University of Sydney Human Research and Ethics Committee.

#### 2.1.1. Scenario 1

First, we conducted experiments on the MSSEG-2016 MS lesion segmentation challenge from MICCAI (Commowick et al., 2018, 2021), containing a totally of 53 cases from 4 different sites, as illustrated in Table 1. In each case, different MR imaging modalities are available, including a FLAIR sequence, a T1 weighted sequence pre and post-Gadolinium injection, a T2 sequence, and a PD sequence. All sequences are co-registered to FLAIR sequences at a similar resolution via rigid registration. In addition, the pre-processing steps are conducted including denoising with the NL-means algorithm, brain extraction via the volBrain platform, and the N4 bias correction. In our experiments, we only use the FLAIR sequence. All experiments were performed in two-fold cross-validation. At each iteration, 3D patches of size 64 × 64 × 64 were randomly cropped from the original FLAIR images, with random flipping and rotation augmentations.

**Table 1:** Details on the scanners for the datasets used in our experiments.

**Client**	**Scanner**	**Site**	**Patients**
**Scenario 1**			
C1	Siemens Verio 3T	University Hospital of Rennes	15
C2	GE Discovery 3T	University Hospital of Bordeaux	8
C3	Siemens Aera 1.5T	University Hospital of Lyon	15
C4	Philips Ingenia 3T	University Hospital of Lyon	15
**Scenario 2**			
C1	GE Discovery 3T	Brain and Mind Center, Sydney	54
C2	Philips Ingenia 3T	St Vincent's Hospital Sydney	21
C3	Siemens Skyra 3T	I-MED Radiology Network Miranda, Sydney	30
C4	Siemens Magnetom 3T	University Medical Center Ljubljana	30

#### 2.1.2. Scenario 2

To further indicate the effectiveness of our proposed framework on the FL MS lesion segmentation tasks in a practical clinical scenario, we conducted experiments using in-house and public multi-scanner MS datasets from 4 different scanners.

Among them, the data from C1, C2, and C3 are obtained from three different hospitals using different scanners. as indicated in Table 1. All the cases are acquired from patients with relapsing and remitting MS, which is diagnosed based on the McDonald 2010 criteria (Polman et al., 2011). Additionally, the disease duration is less than 10 years, with an expanded disability status scale (EDSS) score of less than 4. Each case contains 3D MRI sequences in two modalities, including a T1 sequence without gadolinium administration and a FLAIR sequence. For all the cases, they are acquired under several different geometrics and timing protocols. For the lesion labeling process, the T1 and FLAIR sequences of each case are resampled to a 3 mm slice thickness for accelerated labeling and to provide a common labeling space). First, the automatic Jim 5.0 (http://www.xinapse.com/home.php) is employed to detect and delineate the lesions on the FLAIR images in a semi-automatic manner. For each case, at least two trained neuroimaging analysts at the Sydney Neuroimaging Analysis Centre (Sydney, Australia) confirmed all the segmentations based on the T1 and FLAIR images, to generate final, gold standard reference masks.

To further increase the diversity of the multi-client MS data, we included a public dataset from a new site acquired with a new type of scanner (Lesjak et al., 2018), in addition to the private data from different scanners. This dataset consists of 30 cases imaged from MS patients under 3 different modalities, consisting of a 2D T1-weighted sequence, 2D T2-weighted sequence, and a 3D FLAIR sequence.

For the data usage, we follow the same settings in Scenario 1, where only the FLAIR sequence for each case is employed. To further simulate the practical multi-client scenario, we use the data in their original resolutions, without any registration process. Given the larger scale of the dataset compared with those in Scenario 1, all experiments under these settings were conducted in a three-fold cross-validation manner. During training, the 32 × 32 × 32 patches were randomly cropped from the original MRI data, with the augmentations of flipping and rotations.

### 2.2. Federated MS lesion segmentation framework based on two dynamic re-weighting mechanisms (FedMSRW)

**Figure 2:**
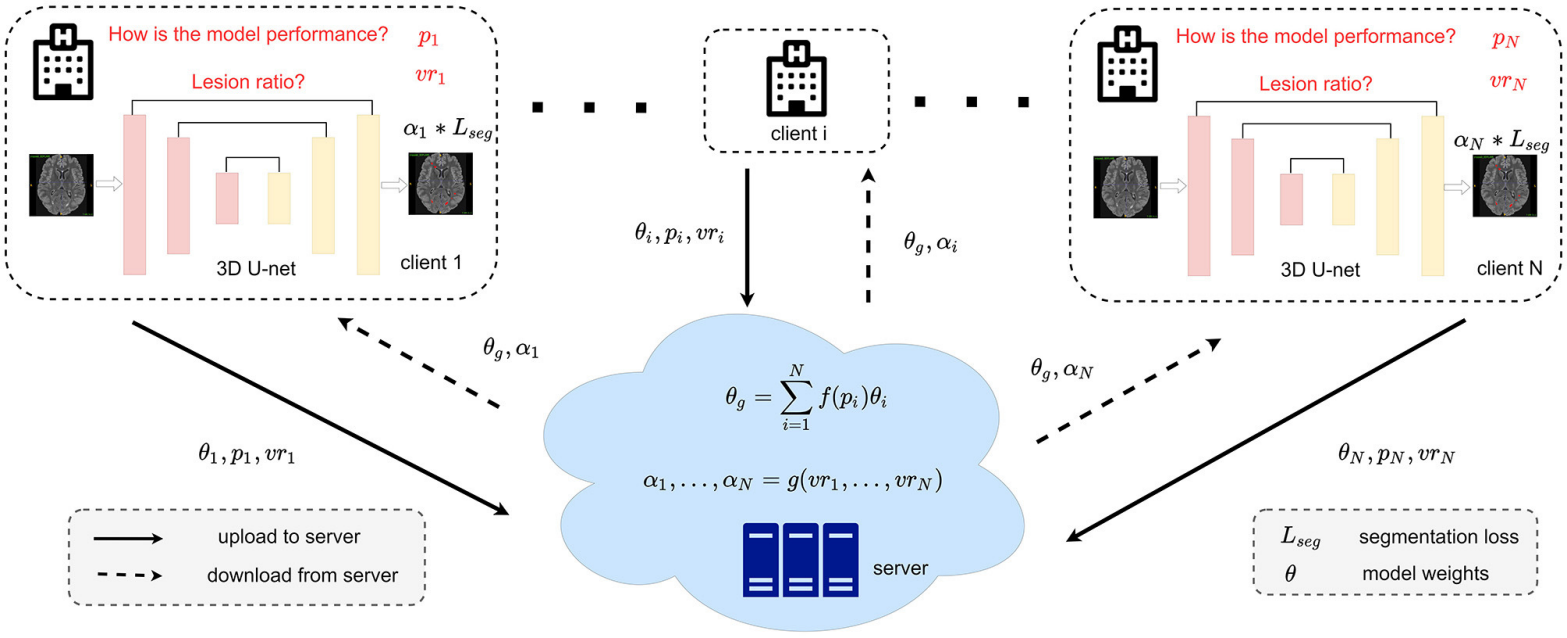
Detailed framework of our FedMSRW method. The *f*(.) for calculating the weighting factors during model aggregation can be referred to Equation (4). The details of *g*(.) for the segmentation task re-weighting are in Equation (6).

The framework of our proposed FedMSRW method is shown in Figure 2. We denote *D*_*i*_ = {*X*_*i*_, *Y*_*i*_}_*i* = 1, 2, ..., *N*_ as the set of MS lesion segmentation datasets from *N* different clients, where *X* and *Y* represent the MR images and the corresponding lesion annotations. In the *ith* client, the local model *M*_*i*_ with the parameters θ_*i*_ is optimized via:


(1) $$\begin{array}{l} L_{i}=\underset{\theta_{i}}{\min}L_{dice}\left(M_{i}\left(X_{i}\right),Y_{i}\right), \end{array}$$

where *L*_*dice*_ is the soft Dice loss function for probabilistic binary segmentations (Milletari et al., 2016):


(2) $$\begin{array}{l} L_{dice}=1-\frac{2\sum M_{i}\left(X_{i}\right)Y_{i}}{\sum M_{i}{\left(X_{i}\right)}^{2}+\sum Y_{i}^{2}}. \end{array}$$

Due to the data distribution differences in multi-client MR images, we establish our proposed FedMSRW on FedBN (Li et al., 2021), which tackles the domain bias issues in FL processes that only require sharing of the model parameters. Based on the assumption that the parameters of the normalization layers in deep learning models represent the domain-specific information (Huang et al., 2018; Chang et al., 2019), FedBN prevents the central model from domain shift by aggregating the parameters in the convolutional layers, while ignoring those in the batch normalization layers. Specifically, each θ_*i*_ can be represented as: $\theta_{i}=\left\{\theta_{i}^{bn},\theta_{i}^{r}\right\}$, where $\theta_{i}^{bn}$ are the parameters for all the batch normalization layers, and $\theta_{i}^{r}$ are those for the rest layers. After collecting the local weights, the central server aggregates model through:


(3) $$\begin{array}{l} {\hat{\theta }}^{r}=\frac{1}{N}\overset{N}{\underset{i}{\sum }}\theta_{i}^{r}. \end{array}$$

Then the central server distributes the updated weights to each local client. At the beginning of the next round of local segmentation training, each *M*_*i*_ is then initialized as ${\hat{\theta }}_{i}=\left\{\theta_{i}^{bn},{\hat{\theta }}^{r}\right\}$.

### 2.3. Central aggregation re-weighting based on the models' segmentation

Due to distinct, client-specific characteristics of both the MRI data and the MS lesions, the difficulty of lesion segmentation tasks differs across clients. To this end, the segmentation ability for the various *M*_*i*_ is different after each round of local training. According to Equation (3), both the low-performance and high-performance models are assigned equal importance during the aggregation process at the central server. This is suboptimal since the local models with inferior segmentation ability influence the updated model from the server and further limit collaborative knowledge learning in FL. A trivial solution to this problem is to adjust the number of training samples for each client, as indicated in McMahan et al. (2017). However, there is no simple, non-biased sample selection mechanism to alleviate the negative effects of the models with inferior performance. Additionally, selecting auxiliary hyperparameters manually in FL would limit the model's robustness.

To this end, we propose an aggregation re-weighting mechanism based on the segmentation performance of each *M*_*i*_ during the training process in the local clients. For each training iteration in client *i*, we define the input data and corresponding labels as *x* and *y*, respectively. The segmentation ability for probabilistic lesion segmentation *M*_*i*_ is measured as:


(4) $$\begin{array}{l} P_{i}=\frac{\sum M_{i}\left(x\right)*y}{\sum y}*\left(1-L_{dice}\left(M_{i}\left(x\right),y\right)\right). \end{array}$$

As indicated in Equation (4), the first item represents the models' confidence in the predicted lesion segmentation. Since the MS lesion region of interest occupies only a tiny fraction (around 1% on average) of the whole brain volume, the confidence value within the true positive lesion regions better reflects the models' lesion prediction certainty relative to traditional methods that measure the models' confidence based on the entropy of the whole prediction map. For the second part (1 − *L*_*dice*_(*M*_*i*_(*x*), *y*)) in Equation (4), the model's segmentation performance is further considered for re-weighting. If the model has a better segmentation accuracy, its attribute during aggregation is upgraded, and vice versa. Finally, the average *P*_*i*_ for all the local training iterations is able to indicate the segmentation ability for the *M*_*i*_. Considering *P*_*i*_, the central aggregation process in Equation (3) is re-formulated as:


(5) $$\begin{array}{l} {\hat{\theta }}_{rw}^{r}=\frac{1}{\sum P_{i}}\overset{N}{\underset{i}{\sum }}\theta_{i}^{r}*P_{i}. \end{array}$$

### 2.4. Local optimization re-weighting based on the lesion volume

Another challenge in FL MS lesion segmentation tasks is the heterogeneity of lesion size across different clients. As indicated in Nichyporuk et al. (2021); Shirokikh et al. (2020), lesions with smaller sizes should be assigned a larger weight during model training. To this end, we further propose to re-weight the segmentation loss functions in each client defined in Equation (1) based on the lesion volume.

For the *kth* round of local training in client *i*, we first calculate the average lesion volume ratio $vr_{i}^{K}$ of all the data samples for training. Specifically, the lesion ratio in each training patch is the ratio of the lesion volume to the brain volume. Compared with only counting the voxel number of lesions, the lesion volume ratio can avoid inaccurate estimations when the proportions of the brain volume in some specific training patches are small. Next, the $vr_{i}^{K}$ is accumulated with the average lesion volume ratio from the previous *k* − 1 round, denoted as *vr*_*i*_. With the increase of *k*, the accumulated *vr*_*i*_ can represent the true lesion volume ratio for the data used during the model training process in each client. In the *K* + 1 *th* round of local training, the segmentation loss in Equation (1) is then reformulated as:


(6) $$\begin{array}{l} L_{i}^{rw}=\frac{\sum_{i}^{N}vr_{i}}{N*vr_{i}}*L_{i}. \end{array}$$

### 2.5. Model training and inference details

The overall training algorithm of our proposed FedMSRW method is indicated in Algorithm 1. In each local client, the lesion segmentation task is trained with a 3D U-Net (Çiçek et al., 2016). During training, we employ the SGD optimizer with a momentum of 0.9, a weight decay of 0.0005, and a learning rate of 0.0002. After every 800 training iteration, the local models are sent to the central server for aggregation. During inference, the model in each client is constructed by the central aggregated convolutional weights and the client-private batch normalization weights.

**Algorithm 1:** Pseudo-code Algorithm for the proposed FedMSRW method.

**Require:** *D*_1_, ..., *D*_*N*_: MS lesion segmentation from *N* clients. In each *D*_*i*_, *M*_*i*_ is the CNN model with the parameters θ_*i*_. P: the number of FL rounds. Q: the number of local training iterations in each round.
1: **for** *p* ∈ [1, *P*] **do**
2: **for** *i* ∈ [1, *N*] **do**
3: Initialize the *M*_*i*_ with the updated global model.
4: Obtain the accumulated lesion volume ratio for *i*.
5: Optimize the *M*_*i*_ via Equation (6) in Q iterations.
6: Obtain the *P*_*i*_ which measures the segmentation ability for *M*_*i*_ by Equation (4).
7: **end for** Aggregate local models in the central servers via Equation (5). Calculate the re-weighting factors in Equation (6).
8: **end for**
9: **return** θ_1_, ..., θ_*N*_

Regarding the data splits, N-fold cross-validation has been conducted on all the experiments to ensure all the cases are evaluated. First, all the images in each client are randomly split into N-folds. For the experiments on each fold, the (N-1) folds are used for training and validation, while the rest fold of the data is employed only for testing. Such a process has been repeated N times and the average segmentation performance of all cases is reported as the final results for each method. During testing, each case is first cropped into patches of the same size as the training inputs. The segmentation results of the patches of each case are then constructed together to form the final segmentation prediction of this case. Our experiment is implemented with PyTorch (Paszke et al., 2017) on 4 RTX 6000 GPU devices with 24 GB memory. The CPU device is an AMD EPYC 7302 16-Core Processor, and the total memory for the RAM is 256 GB.

### 2.6. Evaluation methods for MS lesion segmentation

To evaluate the segmentation performance of our proposed method, we first employed the case-level and voxel-level Dice coefficient, defined as:


(7) $$\begin{array}{l} Dice=\frac{2TP}{FN+2TP+FP}, \end{array}$$

where TP, FP, and FN indicate the number of true positive, false positive, and false negative voxel predictions, respectively. The case-wise Dice score (C-Dice) was obtained by the average Dice score for all cases. For the voxel-level Dice score (V-Dice), we first calculate the total voxel numbers of the TP, FN, and FP predictions for all the testing cases. Next, the V-Dice score is obtained using these accumulated metrics. Additionally, we also evaluated the performance based on the true positive rate (TPR) and false positive rate (FPR) at the voxel level via the accumulated TP, FN, and FP, defined as:


(8) $$\begin{array}{l} TPR=\frac{TP}{TP+FN},FPR=\frac{FP}{TP+FP}. \end{array}$$

## 3. Experimental results

### 3.1. FL MS lesion segmentation performance

In this section, we present the detailed MS lesion segmentation performance under two FL scenarios. Following typical FL methods (Li et al., 2021; Liu et al., 2021a), we also present two common multi-center learning settings as references, including single-client training, and the centralized training. Specifically, the single-client training indicates each client train and test their models locally, without any cross-client communications (**Single**), and the centralized training indicates the model is optimized directly on all the data from all clients (**Central**). For a fair comparison, the **Single** and **Central** methods are implemented via the N-fold cross-validation settings as our proposed FedMSRW under the same data split. The experimental results are shown in Tables 2, 3. Compared with the single client training, our proposed FedMSRW method can achieve stable performance gain under the majority of metrics in both scenarios. Specifically, our FedMSRW method outperformed the single-client training under the case-wise and voxel-wise dice scores, and the voxel-wise true positive rate. In addition, we notice our proposed FedMSRW method can even outperform the centralized training method in the second scenario, without sharing the data across clients.

**Table 2:** Details of the FL MS lesion segmentation results on Scenario 1.

**Metrics**	**Methods**	***C*1**	***C*2**	***C*3**	***C*4**	**Avg**
C-Dice ↑	FedMSRW	68.25	67.32	55.76	69.48	65.20
	Single	68.58	48.92	62.52	61.58	60.40
	Central	70.25	71.07	60.02	70.94	68.07
V-Dice ↑	FedMSRW	75.83	81.44	64.13	75.81	74.30
	Single	78.39	69.70	62.50	63.06	68.41
	Central	78.94	83.82	70.77	77.36	77.72
V-TPR ↑	FedMSRW	67.45	81.35	64.69	70.41	70.98
	Single	70.10	60.60	55.62	49.29	58.90
	Central	75.90	77.97	68.15	75.37	74.35
V-FPR ↓	FedMSRW	13.42	18.47	36.42	17.90	21.55
	Single	11.09	17.98	28.67	12.51	17.56
	Central	17.76	9.37	26.41	20.54	18.52

The direction arrows in the first row indicate the direction of metric improvement.

**Table 3:** Details of the FL MS lesion segmentation results on Scenario 2.

**Metrics**	**Methods**	***C*1**	***C*2**	***C*3**	***C*4**	**Avg**
C-Dice ↑	FedMSRW	52.42	58.90	50.90	52.41	53.66
	Single	55.20	45.69	41.92	58.22	50.26
	Central	55.33	57.63	48.84	48.83	52.66
V-Dice ↑	FedMSRW	64.22	69.48	56.90	58.61	62.31
	Single	63.33	40.28	43.86	69.35	54.21
	Central	64.31	65.99	48.00	55.47	58.44
V-TPR ↑	FedMSRW	64.17	66.02	54.32	45.14	57.41
	Single	58.27	52.53	52.49	62.37	56.41
	Central	56.08	59.74	53.89	40.74	52.62
V-FPR ↓	FedMSRW	35.73	26.67	40.25	16.46	29.78
	Single	30.64	67.33	62.33	21.91	45.55
	Central	24.64	26.29	56.73	13.12	30.20

The direction arrows in the first row indicate the direction of metric improvement.

### 3.2. In comparison with other FL methods

**Figure 3:**
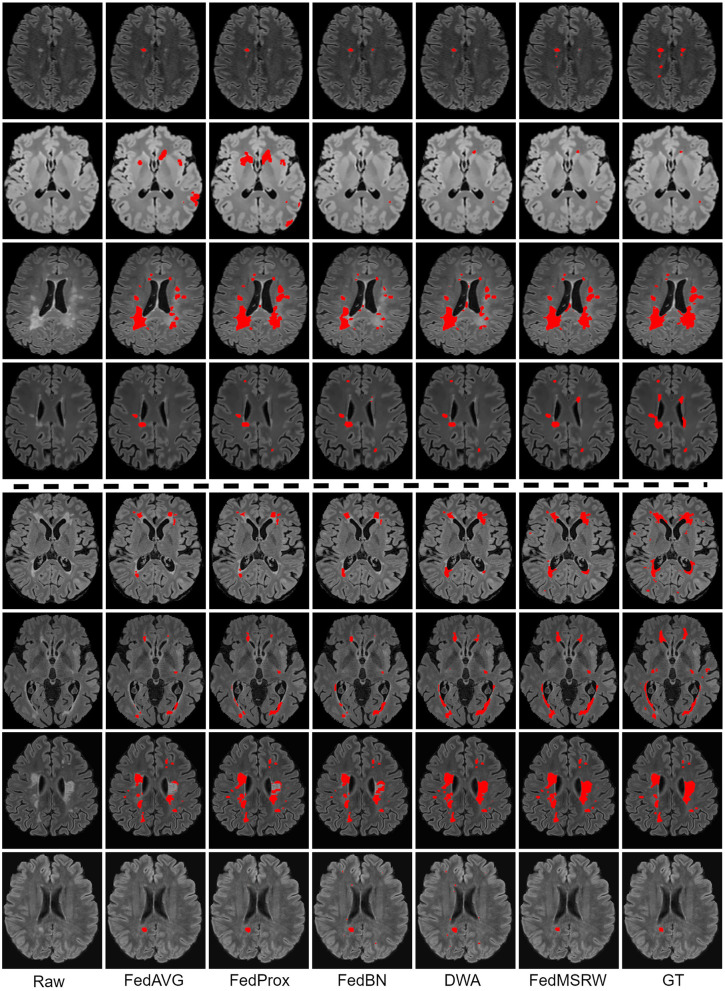
Qualitative results on the comparison FL methods. Lesion masks are overlaid on the original images. The **top** four rows are the visualization for the Scenario 1, and the **bottom** four rows are for the Scenario 2. The examples in all rows are from different patients.

To demonstrate the superiority of our proposed FedMSRW method over other FL methods on FL MS lesion segmentation tasks, we present the experimental results in comparison with typical FL methods, including (1) **FedAvg** (McMahan et al., 2017), a fundamental FL method by central aggregation via averaging of model weights; (2) **FedProx** (Li et al., 2020a), a FL framework introducing an auxiliary regularization mechanism in each client to stabilize learning, (3) **FedBN** (Li et al., 2021), an FL framework which can alleviate the cross-site data distribution bias by ignoring parameters in the normalization layers during aggregation, and (4) **DWA** (Shen et al., 2021), a dynamic re-weighting mechanism for the central model aggregation process based on the changes of the loss functions in each client. For a fair comparison, we re-implement the DWA on the same FL baseline as our proposed FedMSRW method, i.e., FedBN. We also report the results by training within each local client (**Single**), and joint training with the raw data from all clients (**Central**). We maintained the same data split on the N-fold cross-validation for all methods. The experimental results under two FL MS segmentation scenarios are shown in Table 4 and Figure 3.

**Table 4:** Details of the comparison experiments.

	**Scenario 1**				**Scenario 2**			
	**C-Dice** ↑	**V-Dice** ↑	**V-TPR** ↑	**V-FPR** ↓	**C-Dice** ↑	**V-Dice** ↑	**V-TPR** ↑	**V-FPR** ↓
Single	60.40	68.41	58.90	17.56	50.26	54.21	56.41	45.55
Central	68.07	77.72	74.35	18.52	52.66	58.44	52.62	30.20
FedAVG	57.16	56.56	65.59	32.79	47.06	47.78	54.23	41.07
FedProx	59.26	60.72	66.57	29.73	44.06	49.60	55.99	51.03
DWA	63.63	71.56	64.32	19.07	41.68	42.43	**57.70**	59.23
FedBN	64.00	72.87	64.86	**15.87**	49.55	57.47	55.28	35.52
FedMSRW	**65.20**	**74.30**	**70.98**	21.55	**53.66**	**62.31**	57.41	**29.78**

Average values for each metric. Bold values indicate best performance for each case.

### 3.3. Effectiveness on the proposed re-weighting modules

To indicate the effectiveness of our proposed weighting mechanism for the central aggregation (CA) process and local training (LT) process, we present ablation experiments and the results are shown in Table 5. For both two scenarios, we notice that solely employing the CA or LT mechanism can sometimes incur performance drop. However, by jointly incorporating the two re-weighting mechanisms, we can consistently improves the baseline (FedBN) method by a large margin, indicating the effectiveness and robustness of our method on the FL MS segmentation tasks.

**Table 5:** Details of the ablation studies in our experiments.

		**Scenario 1**				**Scenario 2**			
**CA**	**LT**	**C-Dice** ↑	**V-Dice** ↑	**V-TPR** ↑	**V-FPR** ↓	**C-Dice** ↑	**V-Dice** ↑	**V-TPR** ↑	**V-FPR** ↓
✗	✗	64.00	72.87	64.86	**15.87**	49.55	57.47	55.28	35.52
✓	✗	63.95	73.54	68.64	20.61	51.96	61.08	57.70	30.35
✗	✓	64.55	72.29	66.45	20.17	42.39	45.46	**58.74**	61.32
✓	✓	**65.20**	**74.30**	**70.98**	21.55	**53.66**	**62.31**	57.41	**29.78**

The bold values indicate the best performance.

“+ CA” and “+ LT” indicates the FedBN baseline constructed with the proposed central aggregation and local training mechanism, respectively.

### 3.4. Different model design strategies

For deep learning-based medical image analysis models, there can be multiple design selections even under the similar motivation. In this section, we investigate different design choices of our FedMSRW method on the two scenarios. These experiments were conducted on both scenarios and the results are shown in Table 6.

**Table 6:** Results on the effectiveness of our proposed FedMSRW under different model designs.

	**Scenario 1**				**Scenario 2**			
	**C-Dice** ↑	**V-Dice** ↑	**V-TPR** ↑	**V-FPR** ↓	**C-Dice** ↑	**V-Dice** ↑	**V-TPR** ↑	**V-FPR** ↓
Baseline	64.00	72.87	64.86	**15.87**	49.55	57.47	55.28	35.52
Ours-ent	64.14	74.60	69.20	18.77	41.39	46.91	57.36	59.39
Ours-vol	65.38	73.66	66.18	16.74	46.90	53.03	**62.26**	52.21
FedMSRW	**65.20**	**74.30**	**70.98**	21.55	**53.66**	**62.31**	57.41	**29.78**

The bold values indicate the best performance.

First, we replace the model's segmentation confidence in Equation (4) with the entropy map of the whole segmentation predictions (“**Ours-ent**” in Table 6), following typical uncertainty learning methods in medical image segmentation (Yu et al., 2019; Liu et al., 2021b). Equation (4) is then re-formulated as:


(9) $$\begin{array}{l} P_{i}^{e}=-M_{i}\left(x\right)*log\left(M_{i}\left(x\right)\right)*\left(1-L_{dice}\left(M_{i}\left(x\right),y\right)\right). \end{array}$$

Finally, each local model in the central aggregation process in Equation (5) is assigned a weight of $P_{i}^{e}$. In addition, we conducted experiments in which lesion volume was employed for local-level re-weighting on the task learning, referred to as the “**Ours-vol**” method in Table 6. Specifically, the volume ratio *vr*_*i*_ in Equation (6) is replaced by the total number of lesion voxels *v*_*i*_. The results in Table 6 indicate the “**Ours-ent**” and “**Ours-vol**” are less robust than the FedMSRW method, since their performance drops on Scenario 2, while our FedMSRW can improve the baseline on both two scenarios.

### 3.5. Results using different data modalities

For typical deep learning MS lesion segmentation methods (Brosch et al., 2016; Ghafoorian et al., 2017; Valverde et al., 2017; Zhang et al., 2018; Aslani et al., 2019; McKinley et al., 2020; Nair et al., 2020; Isensee et al., 2021; Ma et al., 2022), MR sequences under different modalities are jointly employed to achieve an outstanding segmentation performance. In this section, we have explored whether such implementations are still effective under the FL scenarios. Specifically, we have evaluated the performance of our methods using different MRI modalities. Our experiments are conducted on the MSSEG-2016 challenge, where each subject has five imaging modalities (T1, FLAIR, T2, DP, and GADO). The results are shown in Table 7, where we have presented the results using T1 and FLAIR, and all five modalities. This tables shows that the FL method trained on FLAIR MRI cases can achieve a better performance than on more modalities.

**Table 7:** Details of the experimental results on using different imaging modalities on the MSSEG-2016 dataset.

	**FedMSRW**				**FedBN**			
	**C-Dice** ↑	**V-Dice** ↑	**V-TPR** ↑	**V-FPR** ↓	**C-Dice** ↑	**V-Dice** ↑	**V-TPR** ↑	**V-FPR** ↓
FLAIR	**65.20**	**74.30**	**70.98**	21.55	64.00	72.87	64.86	**15.87**
FLAIR & T1	61.77	71.97	65.89	19.49	63.00	72.12	65.02	18.45
ALL	60.83	71.35	63.57	16.11	62.54	72.60	68.78	22.08

The bold values indicate the best performance.

## 4. Discussion

We have presented here an FL MS lesion segmentation framework, FedMSRW, which includes two innovative re-weighting mechanisms for improved performance of the FL aggregated model. Specifically, a learnable weight is assigned to each local node during the aggregation process, based on its segmentation performance. In addition, the segmentation loss function in each client is also re-weighted according to the lesion volume for the data during training.

In contrast to typical FL benchmark tasks, which assume the disease burden/lesion loads for each client are in the same distribution space (Li et al., 2021), the MS lesion segmentation task is confounded by substantial inter-client lesion heterogeneity / distinctions. For the multi-client MS lesion segmentation dataset, the data distributions for each client are distinct, reflecting variance in hardware and image acquisition protocols. This results in domain bias issues when optimizing the aggregated model on each local client. For MS lesion segmentation task, the foreground objects (i.e., lesions) are almost always small and numerous, with a heterogenous spatial distribution. For specific clients whose MR images generally contain smaller lesions with more noise, it is more challenging for a 3D U-Net to segment lesions accurately. In the first scenario of our work, the MS lesion segmentation experiments were conducted on images the MSSEG-2016 dataset. As shown in Table 4, the performance of the typical FedAvg and FedProx methods is worse than the models solely trained with the data in each specific client, which did not demonstrate the benefit of inclusion of additional dataset through federated learning. Subsequently, the domain shifts incur inaccurate segmentation performance for the FedAvg and FedProx methods. By preserving the domain-specific batch normalization in each client, FedBN can alleviate the issue and improve the locally trained models. With the two proposed re-weighting mechanisms at the global and local levels, our FedMSRW method can further outperforms FedBN.

In the second scenario, FL methods were conducted on the in-house and data and a public dataset, where the data differences across the clients are more distinct, and therefore an overall reduced performance is expected. The experimental results are presented in Table 4. We observed a similar phenomenon as the first scenario, namely that cross-client distribution bias in multi-client MS datasets degrades the collaborative performance of the FedAvg and FedProx, while FedBN achieves much better performance by alleviating the domain bias. However, incorporating the DWA with the FedBN baseline has incurred a severe performance drop. The relatively larger dataset used from each client in the second scenario, which exaggerates client-specific differences in data distribution, may explain this observation. Compared to the limited performance of other comparison FL methods in scenario 2, our FedMSRW can improve FedBN by a large margin, which further indicates the robustness of our proposed method. In addition, Shen et al. (2021) recently proposed an FL method with re-weighting schemes for each local model's training based on the loss value changes. However, its dynamic weighting strategy is sensitive to hyperparameter selections, which lacks robustness. Rather, our proposed re-weighting mechanisms at the global and local levels are effective and simple, without auxiliary hyperparameters. On the other hand, the superiority of our proposed FedMSRW method also indicates its effectiveness.

According to Table 4, FedBN can improve the segmentation performance since it alleviates the distinctions for the cross-client MR images. However, its performance is still limited by ignoring the bias of labeling space on MS lesion segmentation tasks. To solve this problem, we propose a re-evaluation of the weighting mechanism for the central aggregation (CA) process and local training (LT) process. As shown in Table 5, solely employing the CA or LT mechanism incurs an unstable performance gain. In Scenario 1, the LT module marginally degrades the Dice score, and incurs an even larger performance drop in the second scenario. A similar phenomenon has been observed in Shen et al. (2021), namely that re-weighting the training loss functions in each client generates unstable FL performance. For the CA module, this introduces a slight performance gain under all the segmentation metrics. Conversely, in the proposed FedMSRW framework, jointly incorporating the two re-weighting mechanisms consistently improves the baseline (FedBN) method by a large margin, indicating the effectiveness and robustness of our method on the FL MS segmentation tasks. Moreover, our proposed even outperformed centralized training on the voxel- and case-wise dice scores in Scenario 2. This is an important finding to emphasize the superiority and data privacy preserving capability of our proposed FedMSRW method on the MS lesion segmentation data from clinical trials and with large cross-client data distinctions. Figure 3 illustrates a visual comparison of FedMSRW with other methods, which indicates the outstanding segmentation performance of our method from the qualitative perspective.

We further conduct experiments to investigate whether different model design strategy can introduce performance variance, as indicated in Table 5. Due to the severe imbalance of MS lesions in the brain MRI from the clinical practice, utilizing entropy maps incurs inaccurate representations of the model's segmentation confidence, and further degrades the FL segmentation performance in both two scenarios. Therefore, we select the global-level re-weighting mechanism based on the mask probability as defined in Equation (5), due to the consistent performance gain. For the local level re-weighting based on true lesion volume, the “**Ours-vol**” method degrades the segmentation accuracies under all metrics in the two FL scenarios. It is potentially because the inaccurate estimation of the true MS lesion distributions in brain MRI patches for model training. For both the “**Ours-ent**” and “**Ours-vol**” selections, we notice although they can improve the FedBN baseline in the Scenario 1, a severe performance drop has been incurred in the Scenario 2. The potential reasons for this phenomenon are two-folds: (1) each client of the Scenario 2 has more data than those in Scenario 1; (2) the multi-client MS dataset in Scenario 2 is constructed by various datasets from in-house scanners and the public resources, which brings more distinctions for the cross-client data distributions.

Furthermore, as illustrated in Table 7, we have evaluated the performance of our framework using different MRI modalities. We notice that although introducing auxiliary modalities can bring more imaging contrast information for segmentation learning, the models actually suffer from performance drop under the FL settings. A potential reason is that, during the FL process, the data distributions across different clients are heavily distinct, which limits the models' segmentation performance on these clients. In addition, including auxiliary data in multiple modalities also introduce more noise and variences from data processing and registration processes. To this end, the FLAIR only approach in our experiments remains the most effective input imaging modality for FL MS lesion segmentation.

We have conducted a computational complexity study on the aggregation process of the proposed FL methods. Specifically, each aggregation process of our proposed FedMSRW method costs 73 ms, while the baseline FedBN costs 72 ms. Since our proposed method has not included auxiliary trainable modules, no extra parameters are introduced. Given the superior performance of our method indicated in Table 4, we think the auxiliary computational cost of our FedMSRW method is negligible, and our proposed aggregation mechanisms at the global and local levels are effective and efficient.

One limitation of this work is the potential bias for the MS lesion masks. In our experiments, the labeling was done with trained Neuroimaging analysts and tested in simulated FL settings. In real-world FL application scenarios, labeling from different sites can have more variance. In addition, the segmentation models for each client in practical FL might also be different, which limits the usage of the model aggregation mechanisms in our work, as well as the typical FL methods. One of the future directions of this work is to implement our FL method on broad computer vision studies and beyond MS applications, to further explore the utility and generalization ability of the two adaptive aggregating mechanisms. The second future direction is to implement the algorithm on the practical computational platform with multiple servers, since existing FL research studies (e.g., our method, FedAVG, and FedBN) are implemented on a single server for the simulated FL setting. This might overlook some issues due to the distinctions among local servers in real-world scenarios. For example, the performance of each local hardware device varies in practical applications. This brings auxiliary communication costs, although does not affect the segmentation accuracies, since the central server has to wait for every client to finish their local training before aggregation. To address this problem, our third future potential is to facilitate the computational efficiency of the FL framework in practical applications, such as introducing lightweight deep learning models for each local client.

## 5. Conclusion

In this work, we proposed a novel FedMSRW method for MS lesions segmentation under the federated learning settings. Our FedMSRW is featured with global and local reweighting mechanisms to adjust the variance of the MR data and annotations across clients. Extensive experiments in two FL MS lesion segmentation scenarios indicated the superiority of our proposed re-weighting mechanism compared with typical FL methods. The demand for privacy-preserving FL in clinical scenarios heightens the imperative to refine existing approaches. FedMSRW is an important methodological advance for analyzing heterogenous multi-client imaging datasets with FL.

## Data availability statement

The original contributions presented in the study are included in the article/Supplementary material, further inquiries can be directed to the corresponding author.

## Ethics statement

The studies involving human participants were reviewed and approved by the University of Sydney Human Research and Ethics Committee. Written informed consent for participation was not required for this study in accordance with the national legislation and the institutional requirements.

## Author contributions

DL designed the research method, conducted the code implementation, and wrote the draft. MC, DW, ZT, LB, and GZ have been involved in the research design and discussions, and the manuscript revision. YL, KK, LL, and JY have been involved in in-house data processing and annotations. C-CS and AN have been involved in the project management. EK, RS, FC, MB, WO, WC, and CW have been involved in the project supervision, project support, research design, and manuscript revision. All authors contributed to the article and approved the submitted version.
